# Assessment of Perceived Attractiveness, Usability, and Societal Impact of a Multimodal Robotic Assistant for Aging Patients With Memory Impairments

**DOI:** 10.3389/fneur.2018.00392

**Published:** 2018-06-01

**Authors:** Justyna Gerłowska, Urszula Skrobas, Katarzyna Grabowska-Aleksandrowicz, Agnieszka Korchut, Sebastian Szklener, Dorota Szczęśniak-Stańczyk, Dimitrios Tzovaras, Konrad Rejdak

**Affiliations:** ^1^Faculty of Education and Psychology, Institute of Methodology and Psychological Diagnosis, UMCS, Lublin, Poland; ^2^Department of Neurology, Medical University of Lublin, Lublin, Poland; ^3^Centre for Research and Technology Hellas, Information Technologies Institute, Thessaloniki, Greece; ^4^Medical Research Center, Polish Academy of Sciences, Warsaw, Poland

**Keywords:** robotic assistant, service robot, mild cognitive impairments, dementia, acceptability, usability, societal impact

## Abstract

The aim of the present study is to present the results of the assessment of clinical application of the robotic assistant for patients suffering from mild cognitive impairments (MCI) and Alzheimer Disease (AD). The human-robot interaction (HRI) evaluation approach taken within the study is a novelty in the field of social robotics. The proposed assessment of the robotic functionalities are based on end-user perception of attractiveness, usability and potential societal impact of the device. The methods of evaluation applied consist of User Experience Questionnaire (UEQ), AttrakDiff and the societal impact inventory tailored for the project purposes. The prototype version of the Robotic Assistant for MCI patients at Home (RAMCIP) was tested in a semi-controlled environment at the Department of Neurology (Lublin, Poland). Eighteen elderly participants, 10 healthy and 8 MCI, performed everyday tasks and functions facilitated by RAMCIP. The tasks consisted of semi-structuralized scenarios like: medication intake, hazardous events prevention, and social interaction. No differences between the groups of subjects were observed in terms of perceived attractiveness, usability nor-societal impact of the device. The robotic assistant societal impact and attractiveness were highly assessed. The usability of the device was reported as neutral due to the short time of interaction.

## Introduction

The aging population of developed countries forces our societies to embrace change in an effort to provide help to their eldest citizens. The worldwide data (United Nations 2017 report^1^; WHO 2012 report^2^; EU ECHIDATA^3^; ADI 2015 Report^4^; ADI 2016 Report^5^) shows the dramatic increase of the number of the persons suffering from memory impairments among which the dominant cause is Alzheimer's Disease. The common prodromal stage is the mild cognitive impairments (MCI) phase, in which most of the deficits are connected with the memory domain [ICD 10^6^; DSM-V; (1–6)]. The present active person struggles with the performance of daily activities due to increasing memory problems affecting: maintenance of a fruitful social life, household tasks and completing errands (7–14).

The well-known criteria of the cognitive decline evaluation (1, 2) does not always reflect subjectively-reported difficulties in everyday functioning. The increasing number of persons suffering from memory decline which is still in the range of age-associated memory impairments (AAMI) is observed. The aforementioned is connected with the ceiling effect of the screening tools used among persons with a higher education who remain active in many fields of everyday functioning i.e., one who, despite old age, still works or continues their education (15). Such a phenomena is described as the Einstein effect and is connected with a significant decline of the cognitive functions of the individual but not classified as MCI (16). For this group of persons the observed difficulties are interfering with their everyday tasks but due to the developed mnemonic techniques they are still able to function at the average for their age level. Problems are observed if they have to cope under pressure or multitask (17–19).

Similar functioning is observed among persons with diagnosed MCI. Physical and mental tiredness, increased stress level and a need for multitasking negatively influences the capacity of the working memory and therefore the execution of everyday tasks (20). It is worth remembering that for a person with memory impairments most of the activities, even if typically automatic, constantly remain in the loop of active and conscious processing (21–23). Otherwise the less significant steps of the executive action are forgotten or performed inadequately.

Observed aging of the developed countries' societies (United Nations 2017 report^1^; EU ECHIDATA^3^; (7); United Nations 2015 report^7^; United Nations 2017 revision^8^) forces the development of the alternative ways to support their citizens. The majority of their oldest population struggles with the increasing loss of independence due to growing physical impairments combined with memory deficits (WHO 2012 report^2^; ADI 2015 Report^4^; Alzheimer's Association 2014 report^9^). Due to demographic changes observed in developed countries the need for supporting human caregivers with robotic aids is increasing (24–30). Such a way of support has been investigated and developed in Japan where the robotic assistants are used in order to decrease the caregiver's burden with tasks such as lifting, monitoring the patients' activity and providing the company combined with cognitive exercises [RIKEN^10^; (31–34)]. This approach is currently investigated within European countries as well (HOBBIT project^11^; RAMCIP project^12^).

Based on the results of the Executive Summary World Robotics 2016 Service Robots report (Executive Summary World Robotics, 2016^13^) the estimated number of service robots currently in use worldwide is 5,400,000, among which only 4,713 are described as devices for disabled persons (handicap assistance robots). The forecast for 2016–2019 is that the total number of service robots will increase by 42,000,000 new devices out of which 8,100 are going to be dedicated devices for helping in performing everyday tasks at home in a more sophisticated way (robot companions/assistants/humanoids). The service robots can be clustered into a subgroup of socially assistive robots which can be of a service type (performing particular tasks to facilitate everyday functioning) and companion type (serving as entertainment and cognitive stimulation) (35).

**Figure 1:**
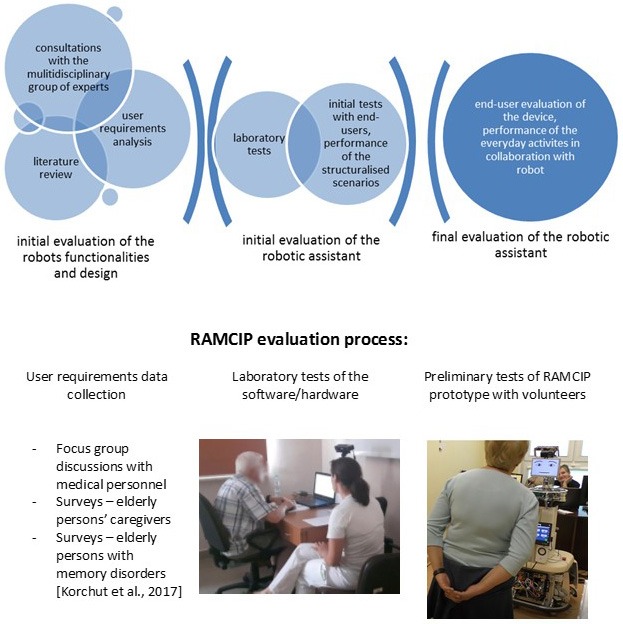
RAMCIP evaluation process.

In current robotic research, user opinion is at the center of attention (36–43). This can be analyzed in two ways: either by multiple tests with the subsequent versions of the device or by examining user requirements followed with prototype testing and later with testing of the final version of the device. The early tests on the rough version of the device as well as the collection of the user requirements are usually performed with the involvement of multiple groups i.e., professionals in the field, end-users and the other interested parties (24, 44–49). The results obtained are introduced into the later design of the robot's architecture. The multistep evaluation procedure monitors the real impact on device evolution and directly moderates the final product's functionalities and outlook. The details of the evaluation methodology are presented in Figure 1.

The theoretical bases for human-robot interaction (HRI) evaluation are relatively recent and originate from research on computer anxiety (50–54) and social psychology (46, 48, 52, 55, 56). One of the first theoretical works designed for the prediction of informational technology acceptance and usage was Technology Acceptance Model (TAM) by Davis (57). The main features that, according to the author, have an impact on the actual usage of the device are: perceived ease of use, perceived usefulness, attitude, behavioral intention to use. Despite the passing years, the newly developed models were using TAM as a core and added new variables to the main model (58, 59). The newest theoretical approach is Unified theory of acceptance and use of technology (UTAUT) by Venkatesh et al. (60) and distinguishes: performance expectancy, effort expectancy, social influence and facilitating conditions as key variables to the acceptance of the device and therefore to actual use of the device. The authors revised existing theoretical models and performed the experimental study to support their approach. The recent work performed by Alaiad and Zhou (61) adds to the model trust, privacy, legal and ethical concerns.

It was observed that in the case of socially assistive robots the social model of interaction can be applied only if the person perceives the robotic assistant as a near-to-human partner of interaction (62–64). The interaction has to be simultaneously set at the cognitive and emotional level. The introduced functionalities such as: the interactive interface, communication based on verbal and non-verbal channels, the proximity adjusted to different task performance, the animated emotional expressions shown by multimodal display and prosody significantly correlate with the length of the voluntary interaction and therefore increase the acceptability of the device (40, 47, 65–68). The accepted level of the robot's anthropomorphism of the device is on the other hand connected with the well-known uncanny valley paradigm introduced by Cheetham and Jancke (69). Not only does the general appearance of the device have an impact on perceived anthropomorphism but so too does the manner of movement, as well as speed (63). The need for complementary implementation of all abovementioned features is a far from trivial task and poses one of the most difficult challenges for designers. On one hand an overly-mechanical device with high anthropomorphic movement and other features is perceived as threatening. But on the other hand an animalistic design combined with the machinelike way of functioning creates a similar sensation. The cognitive dissonance observed in the person negatively correlates with the will of interaction with the device. This may be connected with subconscious application of the social rules of human interactions into HRI at the individual level.

A currently-running project (RAMCIP) Robotic Assistant for MCI Patients at home, whose main aim is to support independent living of the elderly person with memory impairments by providing proactive help in everyday living at home, claims to meet such demands. The RAMCIP project's main objectives are providing the person with the robotic assistant that proactively and discretely monitors the user's actions in order to give a needed level of support to prolong satisfying, independent living at home. The recent trends in demographics, described at the beginning of the section, indicate the need for fast introduction of such a device, that would enhance the human caregiver in his everyday support provided to the elderly patient. The high functioning elderly person with memory impairments, usually with deficits classified as AAMI or MCI, would benefit from RAMCIP due to maintained independence but increased security level. Fast development of the accessible devices, as to price, size and appearance of the robotic assistants gives the chance for prevalent usage of such aid within this age group. RAMCIP's main features have been evaluated in correspondence to the above cited UTAUT model and aims to fulfill the needs of the persons described above.

In the case of the RAMCIP project, the initial evaluation of the robot functionalities and design was performed by the groups of medical professionals (doctors and psychologists), formal caregivers and end-users (patients suffering from MCI/early AD and their caregivers). On the basis of the data analysis, the user requirements were formed (70). The detailed analysis of the RAMCIP design was performed by clinical psychologists and end-users. The required changes in the robot prototype were applied.

The next step evaluation was performed at a laboratory setting. The main software enabling the proper robot functioning was written on the basis of the data gathered during experiments devoted to the main robot functionalities. The integrated solutions and the requirements described in the latter paragraph were implemented into the RAMCIP prototype.

The step-by-step evaluation of the RAMCIP prototype has been performed in accordance to the findings cited in the present chapter. First the detailed research on the particular partial features was performed. The analysis of the users' acceptance of the speech and facial expressions was performed. The proximity ranges were suggested by the clinical psychologists where the uniqueness of the target population was taken into consideration. The general appearance of the prototype was performed as well. The obtained feedback information was introduced in the RAMCIP prototype and later used for the initial HRI tests. The detailed information on the development of the abovementioned solutions are described elsewhere (71).

Based on the findings of the analysis quoted above, RAMCIP's current design and functionalities were introduced. The main area of support covers those most affected by aging and memory impairment, such as: reaction to potentially hazardous events, providing help during cooking, monitoring medication intake, provision of the cognitive stimulation and maintaining a positive mood by helping the user to maintain their social network. In order to facilitate this the robot is equipped with two displays to provide support for multimodal communication. The upper tablet/display, shows the animated emotional facial expression corresponding to the situation and the verbal comments given by the robot. Simultaneously sentences are also displayed at the lower tablet/display in order to minimize potential misunderstandings. The user can communicate with the robot either by voice, gesture or touch by choosing the proper response at the lower display. The iconic representation of particular tasks required by the user, facilitates the (HRI).

Additionally, the robot's elevation mechanism and the dexterous hand allow it to fetch objects desired by the user but in difficult to reach places for an elderly person, such as those which are too high or would require significant bending or stooping. Additionally, the mobile platform allows the robot to navigate autonomously in a domestic environment.

The research questions set during the present study were devoted to verification of the users' requirements described by Korchut et al. (70) implemented into particular HRI scenarios in terms of:

1. Their perceived acceptability measured by: attractiveness, perspicuity, efficiency, dependability, stimulation and novelty of the robotic assistant
2. Perceived usability of the robotic assistant measured by: Pragmatic Quality (PQ), Hedonic Quality Identification (HQI), Hedonic Quality Stimulation (HQS), and Attractiveness (ATT)
3. Establishing the level of the societal impact of the robotic assistant perceived by the participants.

The assessment was focused on the RAMCIP prototype functioning and the level of its perceived acceptability, usability and societal impact by the end-users. The functioning of the RAMCIP prototype user's support had been assessed with standardized questionnaires: User Experience Questionnaire (UEQ) (72–76) and AttrakDiff, as well as a specially-created societal impact survey. The data (UEQ and AttrakDiff) from 9 healthy and 8 MCI participants had been obtained. Ten healthy and 7 MCI patients gave their feedback information in a societal impact survey.

## Methodology

### The recruitment procedures

The participants of the RAMCIP prototype evaluation trials consisted of the elderly volunteers, inhabitants of the Lublin voivodeship. The participants suffering from the MCI were the patients of the Department of Neurology. The participants were not paid for their contribution in the trials.

The information about the upcoming trials was circulated by the local media and the coauthors. The study protocol was positively reviewed by the Medical University of Lublin Ethics Committee (KE-0254/247/2016). The approval for the tests' execution was granted.

The volunteers were assigned the particular dates of the screening visits. The screening consisted of medical and psychological assessment. Based on the results of the examination participants were enrolled to the trials or declined. Prior the screening procedures the informed consent was signed and the purpose of the trials was explained in detail.

### Inclusion and exclusion criteria

The main inclusion criteria were set to include the elderly persons (in the range 55–90 years old, both genders) with and without memory impairments. The criteria of Alzheimer Disease (AD) and MCI proposed by McKhann et al., Albert et al., and Petersen (1–3) were applied. The level of the cognitive functions kept was within the range enabling the relatively mild impaired independent functioning (Mini-Mental State Examination ≥ 20, Clinical Dementia Rating scale ≤ 1, Global Deterioration Scale ≤ 4).

The main exclusion criteria focused on eliminating the persons with conditions reflecting on the cognitive functioning, but not related to the AD nor-MCI such as mental retardation, psychotic syndromes, depression, untreated metabolic disorders, or substance abuse.

The main issue underlined during the recruitment was the ability to declare the informed consent and communication with the personnel.

### The setting and execution of the RAMCIP prototype assessment

The clinical application of the robotic assistant RAMCIP, i.e., the first tests of the HRI, was conducted in the semi-controlled environment at the premises of the Department of Neurology, Medical University of Lublin, Poland. The trials were performed from December 2016 till March 2017. The participants (10 healthy elderly persons and 8 patients with mild cognitive impairments) were performing a number of pre-defined use case scenarios in which typically observed problems in everyday functioning had been addressed. During the scenarios, RAMCIP facilitated users in medication intake, meal preparation and communication with a relative. RAMCIP also reacted to potentially hazardous events. RAMCIP's mobile platform and elevation mechanism enabled reaching high spots and in a proactive manner, supporting in this way the user's activity. Required objects could be fetched by a dexterous manipulation hand. The implemented interface allows the user to communicate in an intuitive and comfortable way with RAMCIP and the external persons.

The proposed use case scenarios (7 in total) were clustered into typical everyday activities such as: cooking, leisure time, medication intake or social interaction. The length of the one-time scenario execution varied:

- Fall detection ~3–5 min
- Assistance in turning off electrical appliance ~10 min
- Assistance upon detection of abnormalities related to electric appliances during cooking ~10 min
- Assistance (proactive/on demand) for fallen objects ~7 min
- Taking medication/food supplements- reminders, brining and monitoring ~15 min
- Proactive bringing of a bottle of water ~10 min
- Proactive communication with relatives and friends ~5 min

During this time the following activities were performed: the introduction to the SubUse case scenario (SubUC) by the researcher, the participant's questions were resolved, and the SubUC execution. The SubUC were run at least twice with each participant.

It was possible to have breaks between the fulfilled scenarios. The scenarios were executed during the organized sessions taking up to 3 h of HRI. During the execution of the activities the researcher was present in the room. After performing of all planned scenarios the participant was asked to assess RAMCIP by fulfilling pen-paper questionnaires. The researcher was available to the participant during the trial execution and the follow-up procedures.

The example of the scenario of the highest importance may be medication intake during which RAMCIP monitors, reminds, and physically helps the user in fulfilling the fixed-in-time activity which is medication intake. The mutual verbal communication is supported by gesture recognition and the execution of the commands by touch at the lower RAMCIP tablet. The robotic assistant initially reminds the user of the set of activities and, if declined, informs the caregiver by sending a text message of the user's reaction. If the user supports the activity but shows high fatigue RAMCIP offers physical help with fetching the pillbox to the user. After performing the medication intake, which is monitored by RAMCIP, the pillbox may be placed back either by the user or by the robotic assistant.

Another scenario of high importance is meal preparation. RAMCIP provides help by monitoring the cooking area and reminding the user about switched-on equipment i.e., cooker. RAMCIP notifies the user if the object is detected on the floor. The scenario is supporting independent meal preparation decreasing most common hazards connected with typical functioning of the elderly person with memory impairments.

RAMCIP's background function is responding to hazardous events such as a fast change in the user's position indicating a fall or increased fatigue detected in the person's gait.

### Statistical methods applied

The data gathered was analyzed with SPSS 22. The descriptive statistics for the demographic, psychological and questionnaire data were performed. The normality of distribution was tested with W Shapiro-Wilk. Depending on its results the parametric (independent *t*-test) or non-parametric statistics (U Mann-Whiney) were applied. The effect size understood as: measure of the magnitude of the effect independent of sample size (77, 78), was verified using d Cohen and the consistency of the results distribution (79) checked with the alfa coefficient.

### Participants profile

During the tests, the psychological and medical assessment had been performed and the general demographic information had been gathered. The normal distribution of the values for age and education has been verified using W Shapiro-Wilk, with which the normality of distribution has not been confirmed. There have been no statistical differences between groups in terms of age, education level and the gender distribution (age *U* = 34, *p* > 0.5, d Cohen = 0.26; education level *U* = 35, *p* > 0.5, d Cohen = −0.21; gender distribution *U* = 37, *p* > 0.5). Both groups' samples consisted of the participants with 11–16 years of education and 60–81 years old. The gender proportion has been balanced within groups (healthy F/M = 7/3, MCI F/M = 5/3) corresponding the gender distribution observed within the population of Alzheimer-diseased patients.

The participants underwent psychological assessment to apply the proper group qualification according to the level of their cognitive functioning and the existence of the memory impairments. The standardized, well-known tools of assessment had been used (Mini-Mental State Examination, Clinical Dementia Rating scale, Global Deterioration Scale) (80–82). The tools of psychological assessment used are standardized and well recognized tests with the Polish language version. The normal distribution of the values for MMSE, CDR, and GDS has been verified using W Shapiro-Wilk, with which the normality of distribution has not been confirmed.

The results obtained by participants in the screening were significantly different in terms of global cognition and ADL scales (MMSE *U* = 1.5, *p* < 0.01, d Cohen = 2.21; CDR *U* = 13, *p* < 0.01, d Cohen = −1.73; GDS *U* = 10, *p* < 0.01, d Cohen = −1.93), which confirms the adequate group selection procedure.

The average healthy participant would suffer from AAMI that do not interfere significantly with his daily activities (CDR median = 0, GDS = 2). Presented by him/her, the level of global cognition is MMSE WS = 27.5 point. The average participant from the MCI group suffers from the memory impairments that interfere with his daily activities (CDR median = 0.5, GDS median = 3) and his/her global cognition level is MMSE WS = 25 points.

None of the participants fulfilled the criteria of depression (Geriatric Depression Scale 2–4 points within all the sample).

### The methods of assessment applied within RAMCIP prototype evaluation

In order to evaluate the RAMCIP attractiveness and acceptance, the UEQ^14^ had been applied (72–76). The scale consists of 26 pairs of adjectives measured on the 7-point Likert scale clustering in six scales (attractiveness, perspicuity, efficiency, dependability, stimulation, and novelty).

UEQ scales should be understood as:

- Attractiveness: Overall impression of the product. Do users like or dislike the product?
- Perspicuity: Is it easy to get familiar with the product? Is it easy to learn how to use the product?
- Efficiency: Can users solve their tasks without unnecessary effort?
- Dependability: Does the user feel in control of the interaction?
- Stimulation: Is it exciting and motivating to use the product?
- Novelty: Is the product innovative and creative? Does the product catch the interest of users? (75, 76).

The questionnaire was filled in by the participant in a paper version. Overall data from 9 healthy participants and 8 MCI patients had been gathered.

In order to evaluate the RAMCIP usability the AttrakDiff Questionnaire^15^ had been applied. The questionnaire consists of 28 pairs of adjectives on the 7 point Likert scale clustering in four subscales.

The AttrakDiff scales should be understood as:

- Pragmatic Quality (PQ)—usefulness and usability of the device,
- Hedonic quality [Identification (HQI) and Stimulation (HQS)]—include emotional needs, such as curiosity, and identification
- Attractiveness (ATT)—is based on the combination of pragmatic and hedonic factors (AttrakDiff Questionnaire^15^).

The questionnaire had been performed as a pen and paper test and later the records were transcribed into the online assessment tool. Overall, the data from 9 healthy participants and 8 MCI patients had been gathered. One participant refused the fulfillment of the evaluation questionnaire.

The questionnaires used for the RAMCIP evaluation have been translated for the purposes of the project. The original authors' acceptance has been obtained for the process of translating the scales to the Polish version. Justyna Gerłowska gained approval for the UEQ Polish translation. The official Polish version is available at www.ueq-online.ogr. Sebastian Szklener gained the approval for AttrakDiff usage for the purpose of the RAMCIP project.

The statistical analysis of the data gathered consisted of the normality of distribution testing with W Shapiro-Wilk. Depending on its results the parametric (independent *t*-test) or non-parametric statistics (U Mann-Whiney) were applied. The effect size and the consistency of the results distribution were checked with the alfa coefficient.

## Results

### Acceptance

The descriptive statistics for the UEQ subscales: Attractiveness, Perspicuity, Efficiency, Dependability, Stimulation and Novelty have been performed. The detailed results may be found in Table 1.

**Table 1:** The distribution of the UEQ subscale values for RAMCIP evaluation.

**UEQ subscales**	**Median (healthy)**	**Median (MCI)**	**Min (healthy)**	**Min (MCI)**	**Max (healthy)**	**Max (MCI)**	**d Cohen**	**Alfa coefficient healthy**	**Alfa coefficient MCI**
RAMCIP Attractiveness	1.66	1.75	0.33	0.83	3	2.5	0.377	0.95	0.61
RAMCIP Perspicuity	2	1.125	0.5	0.75	3	2.75	0.422	0.89	0.6
RAMCIP Efficiency	0.75	1	0.25	−0.5	2.75	2.275	0.33	0.62	0.7
RAMCIP Dependability	2	1.625	0.75	0	3	2.5	0.314	0.75	0.68
RAMCIP Stimulation	1.75	2	0.5	0.25	3	2.75	0	0.8	0.72
RAMCIP Novelty	1.25	0.625	0.5	−0.75	2.5	3	0.587	0.32	0.74

**Figure 2:**
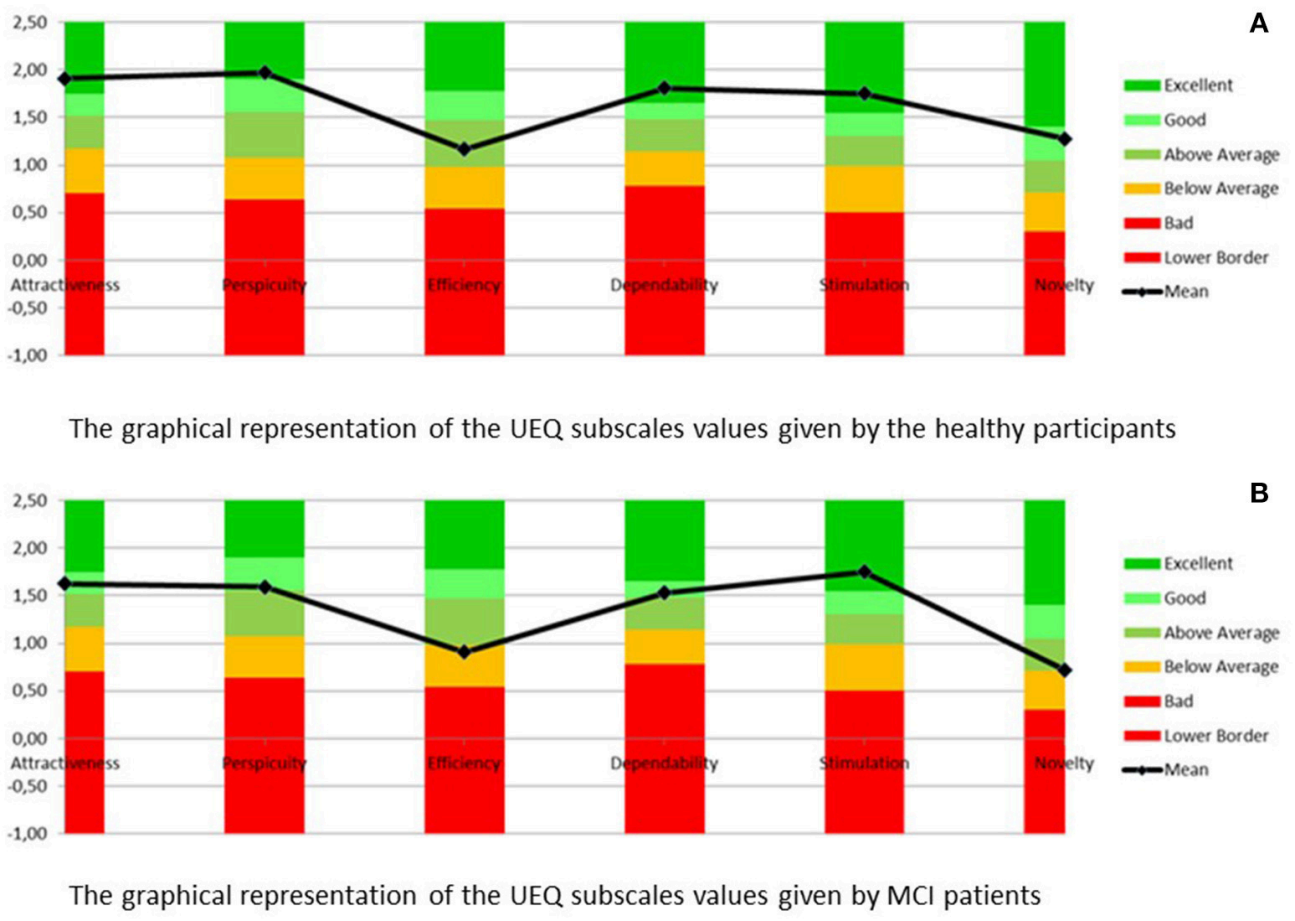
**(A)** The graphical representation of the UEQ subscales values given by the healthy participants and **(B)** the graphical representation of the UEQ subscales values given by MCI participants.

The results obtained from the preliminary RAMCIP evaluation by the users show a high level of acceptance among several UEQ subscales. The corresponding graphs may be seen in Figure 2.

The results show a positive evaluation of RAMCIP within several UEQ scales. No statistical differences between groups in all the subscales have been reported [*t*_(15)_ > −1.23; *p* > 0.23; *U* > 21.5; *p* > 0.17]. The results are depicted in Table 1.

The scales consistency for healthy participants is alfa coefficient >0.75 for all the subscales except Novelty (alfa coefficient = 0.32) and Efficiency (alfa coefficient = 0.62). For the MCI participants all subscales met alfa coefficient 0.6–0.74. Therefore the results should be treated as reliable. The reported effect size is small for all subscales except of Novelty. Due to the low scale consistency it is possible that the reported effect size was biased by the sample size.

The abovementioned results, suggest that RAMCIP meets expectations of its target population in terms of acceptance and that the functionalities that have been implemented are perceived as desired and fulfilled in the design of RAMCIP. The relatively low value of the efficiency subscale may be connected with the developmental level of the project and will be treated as a guideline for the final RAMCIP version.

Attractiveness subscale values may reflect apprehensiveness of the participants and expectations of receiving more compact solution. They as well may be connected with an unrealistic ideal robot image, based on the images known from films. On the other hand, the developmental state of the project allows providing some desired changes.

### Usability

The results obtained do not differ significantly among the groups [*U* > 16.5; *p* > 0.06, and *t*_(15)_ > −0.46; *p* > 0.1] except for: isolating—connective [*U* = 12; *p* = 0.02; d Cohen = 1.61], repelling—appealing (*U* = 14; *p* = 0.03; d Cohen = 1.3), novel—ordinary (*U* = 14.5; *p* = 0.04; d Cohen = 1.25]. The strong effect size has been observed for: pleasant—unpleasant (*U* = 24.5; *p* = 0.29; d Cohen = 0.68), inventive—conventional (*U* = 26.5; *p* = 0.87; d Cohen = 0.61), practical—impractical (*U* = 26.5; *p* = 0.87; d Cohen = 0.49), likable—disagreeable (*U* = 16.5; *p* = 0.07; d Cohen = 1.06), stylish—tacky (*U* = 26.5; *p* = 0.39; d Cohen = 0.58), predictable—unpredictable [*t*_(15)_ = 1.7; *p* = 0.1; d Cohen = 0.82], alienating—integrating [*t*_(15)_ = 1.3; *p* = 0.21; d Cohen = 0.64], brings me closer to people—separates me from people [*t*_(15)_ = 1.17; *p* = 0.26; d Cohen = 0.57], unimaginative—creative (*U* = 19.5; *p* = 0.12; d Cohen = 0.91), innovative—conservative (*U* = 20; *p* = 0.14; d Cohen = 0.86), motivating—discouraging (*U* = 25.5; *p* = 0.34; d Cohen = 0.56).

The results obtained have low subscale values and coefficient values which should suggest reluctance of the participants to give polarized opinions (PQ healthy = 1.32; alfa coefficient = 0.59; HQ healthy = 1.25; alfa coefficient = 0.53; PQ MCI = 0.77; alfa coefficient = 0.61; HQ MCI = 0.67; alfa coefficient = 0.38). The abovementioned result may be also caused by the small group size and diverse opinions.

The results suggest meeting the desired usability performance from a robotic assistant such as the RAMCIP service robot [Pragmatic Quality (PQ), Hedonic Quality Identification (HQI), HQS, and Attractiveness (ATT)]. Due to the small size of the sample, the results should be treated as an indicator but not definite opinion on RAMCIP product.

### Societal impact

For the purpose of the RAMCIP project, the authors in cooperation with sociologists from the University of Warsaw developed an exploratory survey of the opinion of the people who have relevant experience or are currently taking care of people with dementia. The survey was distributed during the tests after HRI among the healthy participants and the MCI patients. The main aim of the survey was to evaluate the assessment tool and also gather additional information about its performance. For the purpose of this study, the results of the perceived potential benefits of the use of the RAMCIP service robot at the MCI patient's home are going to be presented. The particular items with the descriptive statistics may be seen in Table 2.

**Table 2:** The societal impact inventory analysis.

	**Most frequent response**	**Number of participants**	**% of participants**
**THE PERCEIVED INTRUSIVENESS OF THE RAMCIP SOLUTIONS**			
Thanks to RAMCIP I would communicate better with my dependent	Rather agree	7	41.17
RAMCIP's presence would increase my dependent's mood	Rather agree	8	47.05
Thanks to RAMCIP my dependent would become more active in everyday life	Rather agree	8	47.05
RAMCIP's presence would increase my dependent's quality of life	Strongly agree	9	52.94
RAMCIP's presence would increase my dependent's security	Strongly agree	13	76.47
RAMCIP's presence would give me some more free time	Rather agree	9	52.94
RAMCIP's presence would allow me to pick up a job (part-time, remote)	Rather agree	7	41.17
RAMCIP's presence would increase my quality of life	Strongly agree	9	52.94
RAMCIP's presence would increase my dependent's sense of intimidation	Rather do not agree	10	58.82
RAMCIP's presence would increase my dependent's stress level	Rather do not agree	9	52.94
RAMCIP's presence would be intimidating for me	Strongly do not agree	9	52.94
RAMCIP's presence would increase my stress level	Strongly do not agree	8	47.05
RAMCIP's presence would be intimidating for the other family members and guests	Rather do not agree	8	47.05
Robot can help human-caregiver with some caregiving activities	Strongly agree	8	47.05
I cannot imagine communicating with my relatives via robot	Rather do not agree	7	41.17
**BY THE RAMCIP PRESENCE AT HOME, HOW LIKELY IT IS THAT**			
The dependent will feel better somatically and her/his health will improve	Very likely	7	41.17
The dependent will feel better mentally and her/his wellbeing will improve	Very likely	8	47.05
The dependent will feel safer	Very likely	9	52.94
The dependent's cognitive functioning will improve	Quite likely	8	47.05
The dependent will be better in control of everyday activities (medication intake)	Very likely	8	47.05
The dependent will be better in control of her/his finances	Quite likely	6	35.29
The dependent will be more active in everyday life	Quite likely	7	41.17
The dependent will be better in resolving everyday problems	Very likely	10	58.82
The dependent's social interactions within family will improve	Quite likely	9	52.94
The dependent's social interactions will improve	Quite likely	8	47.05

The majority of participants had prior experience as a caregiver of a person with memory impairments or were aware of the amount of help they needed in everyday functioning. The abovementioned was not an intention of the researchers but should be treated as an added value that came to light during performing the surveys. Overall the data from 10 healthy participants and 7 MCI patients has been gathered. One participant refused the fulfillment of the evaluation questionnaire.

The results obtained do not differ significantly among the groups [*U* > 19; *p* > 0.13; and *t*_(15)_ > −0.48; *p* > 0.3]. As mentioned, some of the participants from the MCI group had prior experience as a caregiver as well as being able to relate the questions to their own state. Therefore the results obtained were treated as acquired from the unified group.

The descriptive statistics of the sub-questions within the group have been performed. The results are presented in Table 2.

Based on the results obtained, and in particular the sub-questions generalized into the clusters mentioned below, the participants perceived the implemented solutions as:

- non-obtrusive (47–58%),
- decreasing caregivers' burden (41–53%),
- enhancing everyday functioning of the patient by: facilitating communication 41–47%, positively influencing the mood and behavior of the user 41–47%, positively influencing the functioning of the user 41–52%.

It is worth underlining that 76.5% of participants strongly agreed that the RAMCIP presence would enhance the user's security and 53% that it would benefit his/her quality of life.

## Discussion

The results presented herein suggest the complexity of the clinical application of the robotic assistant and multi-factored acceptability and usability process. The approach taken by the authors resulted in in-depth analysis of the research questions asked.

In terms of acceptability both groups similarly assessed RAMCIP. The robotic assistant was perceived as liked and easy to get familiar with. The results obtained show good and higher assessment of the robotic assistant design. RAMCIP was also perceived as a stimulating and controllable device. Both groups underlined its potential in the everyday rehabilitation of the person with memory impairments. Participants differed in terms of perceived efficiency and novelty of the robotic assistant. It may be connected with the previous experience with new technology and different level of expectations.

In terms of usability the participants were more reserved with their assessment and underlined the necessity of the longer interaction in order to verify the subjective value of the device. The healthy participants were more open with their responses but the results obtained did not differ significantly between the groups. The results obtained show a tendency to perceive a robotic assistant as a useful, pragmatic and hedonic quality device.

In terms of societal impact of RAMCIP the implemented functional requirements presented by Korchut et al. (70) were perceived as met. The high priority functionalities implemented into everyday scenarios were perceived by the participants as significantly beneficiary to the patient's health and wellbeing. The introduced functionalities were perceived as significantly increasing the patient's safety justified precautions.

### Factors influencing the perceived attractiveness and usability

The perceived attractiveness and usability of the device depend on multiple inter- and intrapersonal factors (46, 47, 63, 65, 83–91). The general user's characteristic such as: gender, age, level of self-efficacy, previous experience with new technology have their direct influence on the opinion given (36–38). The Technology Acceptance Model proposed by Davis (57–61, 57) includes them in the group of prior factors and contextual factors. The prior experience gained has significant indirect influence on the final technology's actual usage especially in the oldest age groups (36). The correlation between age, gender, education and the frequency of the modern technology usage has been observed as well (36, 37, 53, 54). The users with the most experience and, therefore, the lowest levels of anxiety combined with the highest levels of self-efficiency are called “silver surfers.” They present the inner motivation to apply the newest state-of-the-art technologies to enhance their everyday functioning. This group is not representative of the general population clustering of the persons with the highest education level, better health, higher incomes and higher levels of cognitive abilities (90). On the other hand, they are most likely to agree to the HRI especially if it involves the tests in the users' home environment. The level of the self-efficacy in the computer usage corresponds with the will of participation in the HRI. In light of such an event it is worth remembering that focusing on the group with the most frequent contact with new IT solutions may falsify the results of the acceptability and usability of the device if generalized to the normal population.

Recent studies underline the importance of the holistic evaluation of the products such as robotic assistants in terms of their attractiveness, acceptability and usability (42). The link between the perceived usability and the acceptability has been shown to influence the final purchase and usage of the evaluated device. The price of the device is important too (36–38), therefore the societal impact should be investigated as well. On the other hand, the observed and widely investigated theory of attribution in social psychology has its implications in the usage of the state-of-the-art technology. The correlation between the opinions of the close circle of friends and relatives and the actual usage of the device has been reported (36, 37).

### Bias observed within the robotic assistants assessments

The literature review performed shows that there are significant differences within the strength of the correlations' influence within the group of experts, students and the target end-users resulting in strong bias (58). The generalization of the results gained from the group of experts may result in misassumption of the importance of the proposed solution. Therefore it is essential to include in the evaluation process the targeted group members. The human-centerd approach applied in the present-day robotic research aims to enhance the chances of positive evaluation of the final product. Reaching the representative members of the target group population, especially in the case of the elder generation, is far from a trivial task. As reported in studies on computer anxiety (53, 54, 89, 90) the elder population is heterogeneous in terms of reported frequency and previous experience with information technologies. On this basis, it is essential to provide a diverse group of users not only in terms of general features like gender, education level and economic status, but in terms of the perceived self-efficiency too.

### Application of the theoretical inclination into RAMCIP evaluation process

All of the above have been thoroughly implemented during RAMCIP prototype evaluation. No significant differences in terms of age, gender and education level has been reported among the end-user groups. The average RAMCIP HRI participant clusters in the group of elder persons (60+ years old) with a medium to higher education level. The proportion of men and women has been balanced within the groups correspondingly to the observed proportions in the population suffering from Alzheimer's disease. The participants' sample differed in terms of IT usage and the perceived self-efficiency in computer usage. The reported diversity did not influence the perceived ease of use of the device. The functionalities available at the prototype were assessed by all the participants at an equal level. The difference in experience with the new technologies may be reflected in the low consistency of the UEQ Novelty subscale.

The societal impact survey's results indicate a high level of perceived usefulness and need of implementing the robotic assistant in the area of the elderly persons with memory problems. Therefore, the results obtained may suggest a potential of a high level of actual use of the device.

## Study limitations

The results described above are based on small groups, therefore the results obtained may be biased by the group size effect. The reported scale consistency suggest medium to high internal consistency. Due to small group size it is not possible to eliminate its possible influence on the values reported. The general tendency reported within the study is that there are no significant differences between the groups in terms of the robotic assistant acceptability, usability, and its societal impact. The size effect reported suggests existing items of medium effect. The further investigation of the evaluation approach taken will be introduced during the pilot trials with the second version of RAMCIP. More participants are going to be invited to the study, therefore the reported limitation herein will be decreased.

## Conclusions

The evaluation of the RAMCIP prototype shows the high acceptance and societal impact of the device at hand combined with the perceived usability in the range of the neutral opinions. The declared readiness for the further participation in the project given by most of the participants was connected with curiosity of the final RAMCIP robot development. Based on previous findings, the attractiveness of the product ranged as valuable by the users, increases with the length of the interaction, which is supported also by the trends observed in the initial tests with the RAMCIP final version. As underlined by the participants the neutral opinion on the usability of the device may be replaced by commitment during longer HRI. The current results may indicate the effectiveness of the approach in the design of the robotic assistant which resulted in the minimization of the level of negative attitude toward the new device. The combination of the desired functionalities and their design fullfills the expectations of the target group.

The current stage of the project is reflected in non-decided responses in the usability scales and efficiency subscales. The needed revisions are going to be introduced in order to provide the user with seamless HRI with the final version of RAMCIP.

On the basis of the literature review and the obtained results, the following recruiting strategy would be implemented in order to provide the most reliable results possible in order to be applicable for the general population of the elderly persons suffering from memory impairments:

- the approach taken so far to evaluate the acceptability, usability and societal impact will be continued,
- the additional structuralized evaluation of the self-efficiency and readiness to new technologies will be introduced,
- special efforts are going to be applied in order to include in the sample the individuals with different attitudes toward new technology.

## Ethical issues

The study protocol was positively reviewed by the Medical University of Lublin Ethics Committee (KE-0254/247/2016). The approval for the tests' execution was granted. The participation was voluntary and anonymous. All subjects gave written informed consent in accordance with the Declaration of Helsinki.

## Glossary

- acceptability- the demonstrable willingness within a user group to employ technology for the tasks it is designed to support (ISO)
- usability- the extent to which a product can be used by specified users to achieve specified goals with effectiveness, efficiency and satisfaction in a specified context of use ISO 9241-11
- societal impact- the potential impact on the individual's socioeconomic situation and the general societal background changes in the case of the device introduction
- socially assistive robot- the robotic device designed for personal use in order to provide stimulation to the human user and facilitate in keeping the social network, maintain, or enhance the level of cognitive functions and quality of life
- HRI—the interaction between human and robot in which both parties remain active and in which the robot is perceived as a social agent. Human and robot share task execution and may interact to synchronize their actions (44)
- digital divide—the significant difference observed among young adults and older adult generation toward new technologies: the frequency of its usage, perceived usability and ease of use
- silver surfer- the person older than 65, extensively using the Internet and the state-of-the-art technology in order to increase his/her quality of life. The technology is perceived as a tool for better everyday functioning, the main purpose of its use is to compensate the observed impairments of the person due to aging processes
- uncanny valley paradigm—M. Mori's theory on robotic acceptance stating that the level of anthropomorphism of the device influences the attitude of the person toward it. The U-shaped curve of increased anxiety is connected with the too mechanical and too humanoid outlook of the robot. The lowest levels of negative emotions provoked in the person were also observed if inconsistencies in the robot's behavior and appearance were minimalized (mechanical with human-like movements or humanoid with machine-like movements).

## Author contributions

JG: substantial contributions to the conception, design of the work, data acquisition, analysis, interpretation of data for the work, drafting the work, and final approval of the version to be published; US, KG-A, AK, SS: contributions to the conception, design of the work, substantial contribution to the data acquisition and final approval of the version to be published; DS-S: contributions to the conception and design of the RAMCIP project; DT; contributions to the conception and design of the RAMCIP project, revising critically data for the work for important intellectual content, and final approval of the version to be published; KR: contributions to the conception, design of the work, revising critically data for the work for important intellectual content, and final approval of the version to be published. All authors agreed to be accountable for all aspects of the work in ensuring that questions related to the accuracy or integrity of any part of the work are appropriately investigated and resolved.

### Conflict of interest statement

The authors declare that the research was conducted in the absence of any commercial or financial relationships that could be construed as a potential conflict of interest.

## Footnotes

^1^United Nations World Population Ageing highlights. (2017) http://www.un.org/en/development/desa/population/publications/pdf/ageing/WPA2017_Highlights.pdf

^2^WHO report: Dementia: a public health priority (2012). http://apps.who.int/iris/bitstream/10665/75263/1/9789241564458_eng.pdf?ua=1

^3^http://ec.europa.eu/health/dyna/echi/datatool/index.cfm?inframe=1#

^4^Alzheimer's Disease International, (ADI) (2015). World Alzheimer Report 2015. The Global Impact of Dementia. An analysis of prevalence, incidence, cost and trends. London. https://www.alz.co.uk/research/WorldAlzheimerReport2015.pdf

^5^Alzheimer's Disease International, (ADI) (2016). World Alzheimer Report 2016. Improving healthcare for people living with dementia coverage, Quality and costs now and in the future, September 2016. London. https://www.alz.co.uk/research/WorldAlzheimerReport2016.pdf

^6^www.who.int/classifications/icd/en/bluebook.pdf

^7^United Nations World Population Ageing report (2015). http://www.un.org/en/development/desa/population/publications/pdf/ageing/WPA2015_Report.pdf

^8^United Nations World Population Prospects: the 2017 Revision (2017). https://esa.un.org/unpd/wpp/Publications/Files/WPP2017_KeyFindings.pdf

^9^Alzheimer's Association (2014). Alzheimer's disease facts and figures. Alzheimer's Dement 2014;10:e47ee92. https://www.alz.org/downloads/facts_figures_2014.pdf

^10^http://www.riken.jp/en/pr/press/2015/20150223_2/

^11^HOBBIT project http://cordis.europa.eu/project/rcn/101982_en.html

^12^RAMCIP project http://www.ramcip-project.eu/ramcip/

^13^Executive Summary World Robotics Service Robots report (2016). https://ifr.org/downloads/press/02_2016/Executive_Summary_Service_Robots_2016.pdf

^14^http://www.ueq-online.org/

^15^http://attrakdiff.de/
